# Assessing site performance in the Altair study, a multinational clinical trial

**DOI:** 10.1186/s13063-015-0653-x

**Published:** 2015-04-08

**Authors:** Nisha Berthon-Jones, Kymme Courtney-Vega, Anna Donaldson, Hila Haskelberg, Sean Emery, Rebekah Puls

**Affiliations:** The Kirby Institute, University of New South Wales, Kensington, NSW 2033 Australia

**Keywords:** Site performance metrics, Altair, Multinational randomised clinical trial, Clinical trial start-up, Participant recruitment, Clinical trial data quality, Laboratory sample quality in clinical trials, Benchmarking performance, Clinical trial performance metrics, Operational metrics

## Abstract

**Background:**

Reviewing clinical trial site performance identifies strategies to control outcomes. Performance across 5 geographical regions (36 sites across Asia, Australia, Europe, North America and Latin America) was investigated in a study that randomised 322 HIV-infected individuals.

**Methods:**

Regional performance was compared using descriptive analysis for time to site opening, recruitment, quality of data and laboratory samples. Follow-up consisted of 10 visits (96 weeks), electronic data collection (EDC) within 7 days of a visit and serious adverse events (SAEs) reported within 24 hours of site awareness.

**Results:**

Median days to site opening was 250 (188 to 266), ranging from 177 (158 to 200) (Australia) to 265 (205 to 270) (Europe). Median days to ethics and regulatory approval was 182 (120 to 241) and 218 (182 to 341) days, respectively. Within regions, time to approval ranged from 187 (91 to 205) days (Australia) to 276 (175 to 384) days (Europe). Time to first randomisation ranged from 282 (250 to 313) days (Australia) to 426 (420 to 433) days (North America).

Recruitment was lower than forecasted in Asia, Australia, Europe and North America at 89%, 77%, 91% and 43%, respectively. The converse was true in Latin America where despite ethics, regulatory and contractual delays, recruitment was 104% of predicted.

Median days to EDC was 7 (3 to 16), ranging from 3 (1 to 16) (Asia) to 13 (8 to 14) days (North America). Median days for initial SAE submission to sponsor was 6 (2 to 20), ranging from 4 (2 to 18) (Latin America) to 24 (5 to 46) days (Australia). Sites took longer to submit final reports, overall median of 28 (7 to 91) days, ranging from 7 days (Australia) to 67 (23 to 103) days (Europe).

**Conclusions:**

Population availability and time to ethics and regulatory approvals influence recruitment; therefore accurate feasibility assessments are critical to site selection. Time to ethics and regulatory approval may not limit site inclusion if compensated by rapid recruitment. Identifying potential delays and methods for reduction can decrease time and costs for sponsors.

**Trial registration:**

Clinical Trials.Gov identifier: NCT00335322. Date of registration: 8 June 2006

## Background

Poor site selection in multicentre randomised clinical trials can result in delayed start-up, unmet target recruitment, poor data quality and/or research integrity, thereby contributing to cost inefficiencies in resource and time allocation. Therefore, tracking a site’s operational performance from a sponsor perspective can identify ways to improve processes for future studies and to enable performance comparison between sites.

Use of performance metrics is two-fold: to improve processes internally (for continuous improvement efforts and future research), and to strengthen relationships with sponsors. Benefits for internal operations include:

- Identifying where processes can be improved
- Identifying where resource allocations can change
- Managing the workload effectively across staff
- Establishing performance benchmarks, and
- Providing data-driven rationale to management to request additional resources.

Benefits from the sponsor relationship perspective include:

- Identifying areas of strong competitive advantage, and
- Ability to complete site feasibility questionnaires with accurate data [1].

Metrics can enable comparison of operational performance between sites [2-5] and their use has been adopted by sponsors to demand higher levels of metrics-driven performance [6-10]. Types of metrics include:

- Leading indicators - information that can be acted on immediately to get the trial back on track
- Lagging indicators - information that can be used for future trials or for process improvement efforts
- Cycle-time - measures the time taken to complete a task
- Timeliness - measures whether a particular milestone was met
- Efficiency - measures the amount of resource required to complete a task or set of tasks versus that expected, and
- Quality - measures how well an output from a process meets the requirements of the customer of that process [1].

Examples of site performance metrics include:

- *Metric #1: Cycle Time from Draft Budget Received from Sponsor to Budget Finalisation*: the time (days) between the date that the first draft budget is received and the date that the sponsor sends approval of the budget. Long cycle times can signal the site to identify areas where the process is being delayed and ways to improve the process. Sites that have short cycle times for this metric can use the information to demonstrate their responsiveness to sponsors [11]
- *Metric #2: Cycle Time from Institutional Review Board (IRB) Submission to IRB Approval*: the time (days) between the dates of initial submission to the ethics committee and the protocol approval. If the site is experiencing delays in receiving ethics approval, process improvement should be investigated between the site and ethics committee. If the site approves new trials in a timely fashion, sponsors are more likely to provide repeat business. Ethics approval is one of the first milestones in the life cycle of a clinical trial and the variability between sites at this step is beneficial; therefore, a good track record for this metric is the site’s advantage when promoting abilities [11]
- *Metric #3: Cycle Time from Full Contract Execution to Open to Enrolment*: the time (days) between the date of an executed contract and the date subjects may be enrolled. Recruitment is a significant challenge across the industry, and as with other metrics, long cycle times could indicate that the site should try to identify areas where the process is being delayed. The metric should be tracked over time to verify if any changes have had a positive impact. If the site’s performance for this metric is good, it should be used to leverage during budget negotiation. Sites have more time to enrol subjects if they are able to get protocols open to recruitment faster. Sites with a history of good performance for this metric will be selected first for future trials [11]
- *Metric #4: Volumetric - Number of Active Protocols*: the number of protocols that are not in long-term follow-up with one of the following statuses: Open to Recruitment, Closed to Recruitment, or Suspended within a given month. This metric determines the site’s clinical research operations capacity and efficiency [11]
- *Metric #5: Volumetric - Number of New Subject Recruitments*: the number of subjects recruited during a given month. This metric measures the site’s ability to deliver on recruitment targets. Sites with a history of successfully meeting recruitment targets are selected first for new trials [11].

Recruitment into multicentre, randomised clinical trials is often slower or more difficult than initially expected, with many trials failing to reach their planned sample size within the originally envisaged timeframe and funding [6,12]. Consequently, trials often require extended recruitment periods supported by additional funding [6,7,13]. Effective and timely participant recruitment is therefore essential for the successful completion of a trial and generation of a valid result, as prolonged or inefficient recruitment can have adverse scientific, economic and ethical consequences [6,7,12].

During site selection, sponsors request feasibility information on how a site performed on similar studies; that is, number of enrolled subjects versus what was contracted. According to the Tufts Centre for the Study of Drug Development, two thirds of sites do not meet patient enrolment requirements of a given study [11]. This metric was used by Winship Cancer Institute at Emory University to implement positive changes as data showed that studies that recruit the first patients slowly are less likely to be successfully completed due to inadequate overall recruitment. Hence, Winship enhanced its closure policies to limit the time and effort wasted on trials that were unlikely to be successful. Changes implemented included stricter guidelines related to recruitment; for example, new trials must reach at least 25% of their target recruitment during the first 6 months or be subject to closure. After 1 year, the Winship Cancer Institute was able to demonstrate positive and measurable changes in the performance of their trials.

There is limited published international, multicentre, randomised clinical trial data in this area. One study outlined the use of benchmarking clinical trials conducted in Europe [8], evaluating sites in terms of goals:

- Temporal - number of days to obtain review approvals to the first subject visit
- Quantity - number of subjects a site can enrol
- Quality - number of queries per case report form (CRF) [8].

The study concluded that benchmarking provided an effective tool for improving data quality, reducing time to market, and declining development costs of international, multicentre clinical trials. Furthermore, a matrix of performance measures was established to evaluate site compliance for the South African National Defence Force-sponsored Project Phidisa [8]. The primary aims of benchmarking in this setting were to improve data quality and increase subject safety. Parameters were evaluated and reported quantitatively, measured against a target standard. The outcome of the assessment provided tangible results that were used to target corrective action in areas needing improvement, not only at the site level but also within the various sections or departments that supported the trial.

Operational data can be tracked within a clinical trial management system (CTMS) or on a spreadsheet; however the challenge lies in analysing and interpreting the data in a way that leads to beneficial change. Therefore, careful consideration is required when setting up a study to ensure all required operational metrics are defined, and can be captured appropriately.

The aim of the current exploratory analysis is to describe regional performance in the Altair study using operational metrics relating to start-up, recruitment, data collection, and stored samples, to plan efficient implementation of future studies.

## Methods

### Study design of Altair

The Altair study, sponsored by The Kirby Institute, University of New South Wales, Australia, has been described previously [14]. Briefly, treatment-naïve HIV-infected individuals were randomised in equal proportions to receive tenofovir/emtricitabine (TDF 300 mg once a day (qd)/FTC 200 mg qd) with efavirenz (EFV 600 mg qd) (Arm I), ritonavir-boosted atazanavir (r/ATV 100 mg/300 mg qd) (Arm II) or zidovudine/abacavir (ZDV 250 mg or 300 mg twice a day (bd)/ABC 600 mg qd) (Arm III). The intention-to-treat (ITT) population consisted of 322 randomised participants who received at least 1 dose of study medication and 1 follow-up visit [14].

Thirty-six sites in 15 countries (Argentina, Australia, Canada, Chile, France, Germany, Hong Kong, Ireland, Israel, Malaysia, Mexico, Singapore, Taiwan, Thailand, and United Kingdom) participated in this study. The sites represented 5 regions including Asia (n = 5 sites), Australia (n = 10), Europe (including Israel) (n = 7), Latin America (n = 11), and North America (n = 3). Ethical approval was received at all sites, although local regulatory authority approval was not required in Australia, North America and Israel. Moreover, public hospitals in Australia, Ireland and United Kingdom underwent governance evaluation of the protocol. Prior to study conduct, written informed consent was obtained from each participant. A list of ethics committees that approved the Altair study is provided in Appendix.

TDF/FTC was supplied as the fixed-dose combination (Truvada) to all sites after packaging and labelling of Gilead clinical trial supply by ALMAC Clinical Services, Craigavon, Northern Ireland. The drug was not registered or commercialised at study initiation in Chile, Hong Kong, Malaysia, Singapore, Taiwan and Thailand.

There were 10 study visits during 96 weeks of follow-up. The Oracle Electronic Data Capture (EDC) platform (Oracle, North Ryde, NSW, Australia) was used for data collection. Data entry was required within 7 days of the study visit and serious adverse events (SAEs) reported to the sponsor within 24 hours of site awareness.

Randomised Altair study patients provided informed consent before permitting storage of plasma and buffy coat samples at 10 time points. At week 0 or baseline, a buffy coat was collected for DNA isolation with the aim of conducting future investigations related to HIV disease. Furthermore, storage of samples for future genomic testing occurred depending on patient consent. Samples were shipped to a central facility (St. Vincent’s Centre for Applied Medical Research, Sydney, Australia) once the final visit (week 96) was concluded. Paper inventories were requested at week 48 and prior to shipping at week 96. The number and quality of samples were interrogated upon receipt in Sydney and were compared by region.

### Statistical analysis

A descriptive analysis of the operational metrics listed below evaluates regional performance across Asia, Australia, Europe, Latin America, and North America:

- Start-up:
  - Time from protocol release to ethics and/or regulatory submission and approval
  - Time from protocol release to First Participant Randomisation (FPR) and Last Participant Randomisation (LPR)
- Recruitment:
  - Time from site opening to FPR
  - Time from FPR to LPR
  - Actual versus estimated recruitment
- Data collection:
  - Time from actual visit to EDC initiation
  - Time from EDC initiation to completion
  - Number of missing values per participant
  - Number of queries opened per participant
  - Number of missed visits per region
  - Number of SAEs reported
  - Time from SAE occurrence to initial report
  - Time from initial SAE report to final report
- Storage samples:
  - Number of plasma samples collected versus protocol-mandated samples to be collected
  - Number of buffy coat samples collected versus protocol-mandated samples to be collected
  - Quality of laboratory samples collected

## Results

### Start-up

**Figure 1:**
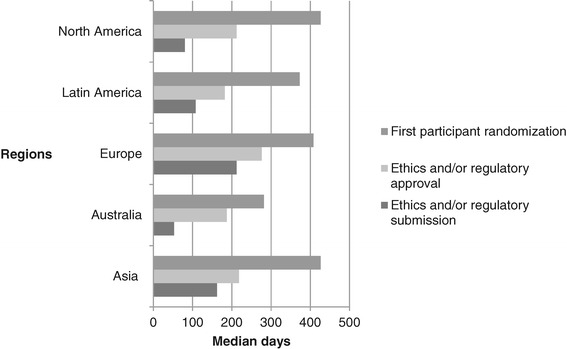
**Characterisation of start-up ethics and/or regulatory by region.**

Median days from protocol release to site opening was 250 (188 to 266), ranging from 177 (158 to 200) (Australia) to 265 (205 to 270) (Europe). Figure 1 is a regional characterisation of the median time in days to submit the protocol to local/regional ethical and/or regulatory authorities, receive approvals, and randomisation of the first participant. By region, sites in Australia took the shortest time to submit and receive ethics approval for the protocol and randomise the first participant. Recruitment of the first participant in Europe, Asia, Latin America and North America took approximately 12 to 14 months from protocol release.

### Recruitment

**Figure 2:**
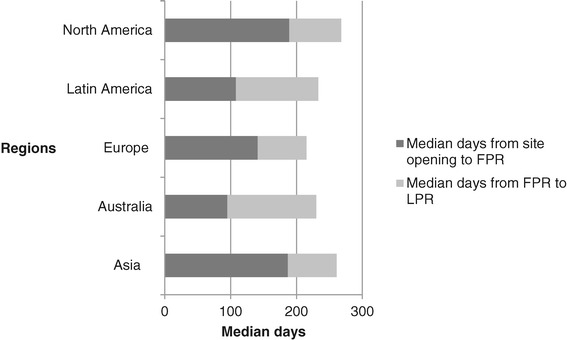
**Recruitment metrics - time from site opening to First Participant Randomisation (FPR) and Last Participant Randomisation (LPR) by region.**

Figure 2 shows the time from site opening to FPR and LPR. Following approval, FPR was rapid in Australia, Latin America and Europe but longer in Asia and North America. Once the FPR visit was conducted for each region, sites in Asia, Europe, and North America had their LPR 2.5 months later. Australia and Latin America completed recruitment in a minimum of 4 months; the shortest recruitment period as measured by the time from site opening to LPR. The recruitment period for Europe and Asia was 8 months and 9 months for North America.

**Figure 3:**
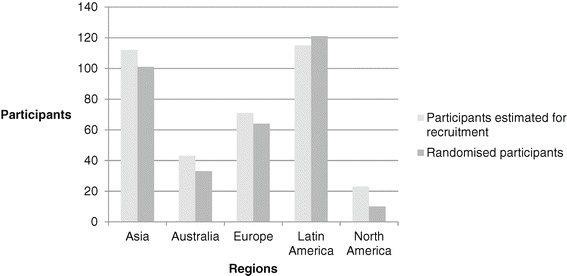
**Actual versus estimated recruitment by region.**

Figure 3 demonstrates participants estimated for recruitment and total randomised. The highest enroller was Latin America, followed by Asia, Europe, Australia and North America. Actual recruitment was lower than estimated in Asia, Australia, Europe and North America, although Latin America (104%) exceeded the estimated number.

### Data collection

#### Data management

Table 1 demonstrates that EDC was slowest in North America (median 13 days), followed by Europe. Sites in Asia, Latin America and Australia started entering data quickly. All sites completed data entry within 14 days and sites in Australia had the longest opened queries per participant (median 2 days), with the other regions responding to queries on the same or next day.

**Table 1:** **Electronic Data Capture (EDC) and serious adverse event (SAE) reporting**

	**Sites**	**Total participants randomised**	**Mean participants randomised per site**	**Median number of queries opened per participant**	**Median number of missing values per participant**	**Median number of missed visits per region**	**Median days from actual visit to EDC initiation**	**Median days from EDC entry to completion**	**SAEs reported per 10 participants**	**Median days from SAE occurrence to initial SAE report**	**Median days from initial SAE report to final, accepted SAE report**
Asia	5	101	20	25	22	0	3	9	2	8	37
Australia	10	33	3	35	26	1	5.6	14	2	24	7
Europe	7	64	9	37	53	1	8.7	11	2	5	67
Latin America	11	121	11	34	15	1	5.7	17	1	4	8
North America	3	10	3	21	26	1	13.2	9	0	0	0

There were a total of 50 missed visits during the course of the study. The median number of missed values per participant was highest in Europe and lowest in Latin America. The remaining three regions had a similar numbers of missed values per participant.

The median number of queries raised per participant was lowest in North America; Asia followed closely behind and the remaining regions reported similar values.

### Serious adverse event reporting

There were no SAEs reported in North America (Table 1). Asia, Australia and Europe reported 2 SAEs per 10 participants, whilst Latin America only reported 1 per 10 participants. Reporting timelines of the initial SAE report ranged from 4 to 24 days and 8 to 67 days for sites to submit the final SAE report.

One unreported SAE was identified in Europe during a site monitoring visit conducted by the project team.

### Storage samples

The expected number of buffy coat and plasma samples for collection during the study was 30,800. Overall, 82% of protocol-mandated plasma samples were received at the central laboratory; ranging from 79% (Asia) to 99% (North America) (Table 2). The proportion of buffy coat samples received was 144%, ranging from 110% (Asia) to 179% (Australia). All sites therefore collected additional buffy coat samples at week 0 that were not required by the protocol. For all regions except Asia, sites failed to collect plasma stored samples even though the participant attended the visit. In Europe, Latin America and Australia, storage samples were not collected at 7, 5 and 5% of the visits attended, respectively.

**Table 2:** **Stored samples mandated by the protocol and received at the central laboratory for testing**

**Region**	**Buffy coat samples**		**Plasma samples**		
	**Protocol- mandated buffy coat samples to be collected**	**Actual buffy coat samples collected**	**Protocol- mandated plasma samples to be collected**	**Actual plasma samples collected**	**Proportion of visits where plasma samples were not collected as required (%)**
Asia	203	224	4,545	3,582	0
Australia	66	118	1,485	1,314	5
Europe	130	199	2,880	2,293	7
Latin America	196	317	4,365	3,607	5
North America	20	25	450	444	1

Furthermore, one site sent whole blood and another four sites sent serum samples that were not on inventories and were discarded by the central laboratory on arrival as the samples were not protocol-mandated. Some of the errors were procedural, where sites consistently collected the incorrect number or type of samples. These errors were not identified in the paper inventory until receipt of samples at the central laboratory.

## Discussion

In the current exploratory analysis, we focused on site performance in terms of speed of start-up, quantity of recruitment, quality of data and stored samples. Given the nature of the paper, ethics approval was not required.

### Start-up

The Altair study commenced in March 2007 and was recruited in 18 months at clinical sites after protocol release. Prior to this study, the Kirby Institute had 6 years of conducting similar clinical trials, although had only previously worked with 50% of the sites participating in Altair.

Due to the study design and nature of regulatory guidelines, sites in Australia, Hong Kong and Taiwan did not require approval from the regulatory authority. Therefore, delays in start-up were due to receipt of ethics and governance approval at hospital-based sites, executed site contracts and indemnities. However, Australia did not require translated documents, thereby contributing to the shortest time between site opening and FPR. The protracted process of obtaining ethics, governance and regulatory approval [15] across countries in Europe significantly delayed FPR by 13 months from protocol release. Randomisation of the first participant in Latin America and Asia was delayed due to establishing site contracts and import permits for study drugs. Importation into Latin America is often difficult, with a challenging clinical trials regulatory environment [16,17] and changing customs requirements. Study drugs underwent re-labelling due to a request from the German regulatory authority, BfARM, delaying delivery to German sites. Obtaining an adequate level of insurance in countries such as Germany and Argentina also contributed to ethics approval delays as sites requested the use of a local insurance provider. Moreover, it was the first time that we were collaborating with certain sites in Canada and Europe and contractual and indemnity negotiations were prolonged from both directions. Nevertheless, sites in all regions were able to recruit quickly once open, recruiting patients within an average of 3 to 4 months. The large number of sites included contributed to meeting the samples size required to power the study.

Study documents requiring certified translation included the protocol, synopsis, participant information and consent forms, contract, indemnity, insurance certificate, questionnaires related to depression, anxiety and stress and quality of life. Documents for Asia were translated into Thai, Bahasa Melayu, Simplified Chinese and Traditional Chinese. Other translations included French, German, Hebrew, Russian, Amharic and Spanish for sites in Europe (including Israel), Latin America and North America. Document translation contributed to the submission delays in these regions [18,19] and the first participant was not randomised until just over a year after the protocol was released to Latin America, Asia, Europe and North America. This measure indicates that the feasibility assessment must capture the required languages to request translation of study documents as soon as they are finalised and prior to protocol release. Furthermore, quotes from translation services using draft study documents should be obtained during study setup to reduce time delays.

A limitation of this analysis is insufficient data capturing detailed reasons for delayed ethics and governance submission and subsequent approvals. Delayed ethics submission could be due to late receipt of translated documents from the sponsor, prolonged review of site-specific documents by the sponsor, ability of the site to prepare a high-quality submission package, availability of department heads to obtain signatures on documents, and the study coordinator’s current workload (that is if there are many recruiting studies, then studies in start-up are not prioritised). In addition to the difficulty posed when establishing site contracts that require translation, delayed governance submissions could also be due to prolonged budget negotiations between the site and the sponsor, and can indicate that the proposed budget is insufficient for a site to cover costs. Therefore, during feasibility and site selection, it would be useful to obtain a site’s standard setup costs including:

- Start-up fees for the site, pharmacy and laboratory
- Archiving and close out
- Lead site fees for additional administration
- Ethics and governance review fees for the main study and substudies
- Pharmacy fees for dispensing, destruction, call back, drug transfers
- Institutional overhead

Sites that are yet to develop a schedule of fees for clinical trial services should consider the above factors and conduct a time and motion study for participant visit-related procedures; for example, time taken to conduct informed consent, assess eligibility criteria, medical history and conduct physical examination by the principal investigator (PI).

### Recruitment

Recruitment was lower than pre-study estimations by sites in Asia, Australia, Europe, and North America due to a lower naïve patient population in the latter three regions and a competing naïve study in Australia. Despite multiple delays during start-up, recruitment in Latin America exceeded the estimated total within a 3-month period.

This metric demonstrates the importance of accurate data when sites are completing feasibility questionnaires. In order to estimate the recruitment target and the intended recruitment period, sites should be asked to provide the total number of active protocols, number of active protocols per study coordinator (SC), number of new subject recruitment and screen failures during a given month, number of previous similar studies and respective recruitment, and number of competing studies.

Knowing how much time SCs spend on various study-related tasks is extremely useful and beneficial from both site and sponsor perspectives. It demonstrates where there may be inefficiencies and room for process improvement, allows improved planning, and enables accurate budgeting for the time and effort required to conduct a study. Documenting work effort requires staff to log their time, whether in a spreadsheet, a CTMS, or other system. With complete and up-to-date logs filled out, sites can view a detailed report of staff time across activities and protocols. Therefore, a site should invest in calculating an average full-time equivalent allocation to ensure staff is neither overburdened nor under-utilised.

Other factors that may impact recruitment at a site may include the process of identifying suitable participants (for example, patient database and referrals) and methods of effective advertising.

Following this analysis, the Kirby Institute implemented a more rigorous feasibility questionnaire to be completed during site selection for future studies.

### Data collection

Only ten participants were randomised in North America although initial data entry following the visit was delayed by a week. In contrast, sites in Asia and Latin America that randomised over 100 participants began data entry within a week after the visit. The number of missing values per participant varied largely from 15 in Latin America to 53 in Europe; however, the number of queries opened per participant remained fairly consistent across regions. The rate of missed visits was very low across all regions.

The majority of the reported SAEs (83%) were hospitalisations, hence extending the reporting timelines between the initial and final reports. There were no SAEs reported from the North American region and most (63%) were from Latin America and Asia, corresponding to the proportion of global recruitment. A total of 63% of the SAEs reported from Thailand were hospitalisations reflecting the current environment for receiving medical treatment covered by health insurance. Strict confidentiality and privacy laws surrounding access to medical records from local hospitals also influenced timelines for completing follow-up SAE reports from this region.

Prolonged data entry, a high number of missing values and missed visits indicate that site staff may require additional protocol and data entry training. Moreover, the SC’s current workload may also be represented within these measures; that is if the workload is high, there is less time to focus on data quality.

The Kirby Institute implemented weekly remote monitoring of data entry for all future studies to ensure improved data quality and integrity.

### Stored samples

Clinical trials of immunologic therapies provide opportunities to study the cellular and molecular effects of those therapies and may permit identification of biomarkers of response. When the trials are performed at multiple centres, transport and storage of clinical specimens become important variables that may affect blood and tissue specimens [20,21]. There were many errors resulting from shipment at trial completion in the Altair study, highlighting the need for better laboratory oversight by site staff.

Over-**c**ollection of buffy coat samples and under-collection of plasma samples identified a need for additional laboratory training during the lifetime of the study; however, as paper inventories were utilised to document sample collection and shipments to the central laboratory occurred at study closure, the sponsor was not alerted to this problem in real-time. Furthermore, the extra buffy coat samples resulted in additional shipping costs borne by the sponsor, and the smaller quantity of plasma ensured fewer samples to conduct future investigations in HIV disease.

The outcome from this analysis proved a need to improve stored sample processes for future studies using several strategies such as improving communication with local laboratory staff, increasing the number and quality of laboratory monitoring visits and greater frequency of shipping to the central laboratory (even if incurring more expense). As a result of the Altair study sample shipments, The Kirby Institute studies now employ an electronic inventory to allow real-time monitoring of the type and number of samples collected by sites. This has allowed early identification and resolution of errors prior to shipment.

## Conclusions

The proposed strategy for comparing performance of service providers in clinical trials was intended to help interpret differences due to factors affecting start-up, recruitment, data and storage sample collection across regions. It was hoped to provide metrics to establish compliance for future studies in order to meet required timelines and improve cost-efficiency.

Multicentre randomised clinical trials that have been conducted since the Altair study have focused attention on site selection strategies tailored specifically to address the target population required for the protocol, as well as attention to recruitment, data and stored sample collection. The site selection process itself is dependent on multiple factors in addition to the quantity of suitable and available participants at the site and PIs are encouraged to be realistic in their recruitment forecasts. In an attempt to reduce cost and time inefficiency, some studies implemented a cut-off on recruitment estimated by sites and others targeted sites that have a good track record in conducting large, randomised clinical trials with known PIs. The Kirby studies also commenced tracking of recruitment more closely, and maintained frequent communication with site PIs and SCs to identify ways to improve recruitment. Remote monitoring of data collected occurred weekly to ensure data entry was timely, missing values were not a recurring problem, and any missed visits were followed up with the site to confirm the reason. Stored samples are being tracked via an electronic inventory to ensure the correct number and type of samples are being collected.

It was apparent with the Altair study that certain regions are more worthwhile than others in terms of cost efficiency and timeliness of recruitment. For example, sites in Asia and Latin America took longer than sites in Australia to commence enrolment, however participant recruitment and data quality in these regions compensated for the initial delays. Moreover, sites in Asia excelled in data quality and storage sample collection relative to other regions.

## Appendix

### List of ethics committees that approved the Altair study

- CAICI, Argentina
- Hospital Central, Argentina
- FUNDAI, Argentina
- Hospital Alvarez, Argentina
- Hospital Alende, Argentina
- Hospital Italiano, Argentina
- Hospital Ramos Mejia, Argentina
- Hospital Posadas, Argentina
- Hospital Rawson, Argentina
- The Alfred, Australia
- St Vincent’s Hospital, Australia
- Sydney South West Area Heath Service, Australia
- The Royal Melbourne Hospital, Australia
- Westmead Hospital, Australia
- Royal Perth Hospital, Australia
- St George Hospital, Australia
- University of Calgary, Canada
- Toronto General Hospital, Canada
- Fundacian Arriaran, Chile
- Hospital Saint-Louis, France
- J.W. Goethe Universitat, Germany
- Medical Group Practice, Germany
- Queen Elizabeth Hospital, Hong Kong
- Mater Misericordiae University Hospital, Ireland
- Rambam Healthcare Campus, Israel
- University of Malaya, Malaysia
- Instituto Nacional de Ciencias Medicas y Nutricion Salvador Zubiran, Mexico
- NHG Domain Specific Review Board, Singapore
- Taiwan University Hospital, Taiwan
- Thai Red Cross-AIDS Research Centre, Thailand
- Chelsea and Westminster Hospital, United Kingdom
- NHS, United Kingdom

## Abbreviations

bd: Twice a day
CRF: Case report form
CTMS: Clinical trial management system
EDC: Electronic data capture
EFV: Efavirenz
FPR: First participant randomisation
IRB: Institutional review board, ITT, intention-to-treat
LPR: Last participant randomisation
PI: Principal investigator
qd: once a day
r/ATV: Ritonavir- boosted atazanavir
SC: Study coordinator
SAE: Serious adverse event
TDF/FTC: Tenofovir/emtricitabine
ZDV/ABC: Zidovudine/abacavir
